# Utilizing Novel Assessment and Instructional Methodologies of Trauma for Residents; A Case of Blended Learning in Shiraz Medical School

**DOI:** 10.29252/beat-080101

**Published:** 2020-01

**Authors:** Shahram Paydar, Hossein Akbarialiabad

**Affiliations:** 1 *Trauma Research Center, Shahid Rajaee (Emtiaz) Trauma Hospital, Shiraz University of Medical Sciences, Shiraz, Iran*; 2 *Student Research Committee, Shiraz University of Medical Sciences, Shiraz, Iran*

The coexistence of humans, the environment, and animals may lead to laceration, injury, and death. The earliest anthropological registration of human being violations and human conflicts backs to almost more than 200 thousand years [1, 2]. Nowadays, still, fall and violations are the leading cause of human trauma [3]. Trauma causes more than 6 million people to die annually and results in around one-sixth of the global burden of disease as well as ten percent of overall mortality [4]. 

To make the burden clearer, yearly, trauma kills more people in comparison to human immunodeficiency virus (HIV), tuberculosis, and malaria combined. Around ninety percent of mortalities occur in developing world [5]. These statistics reveal that educating and assessing general surgery residents is essentials. There are a variety of novel and conventional methods to teach and assess general surgery residents on Trauma. First of all, we should know that, based on bloom taxonomy, a competent surgeon should have three domains of competency, which are cognition (knowledge), psychomotor (skill), and effect (attitude). So learners should be trained and assessed in all areas [6]. 

Also, we should be aware that in the emergency room; the patient's presentation and sensitivity require immediate attention, and that is a matter of golden seconds rather than golden hours [7]. Assessment drives learning, and the goal of assessment “should be for learning,” not "assessment of learning" [8]. Unfortunately, current assessment methodologies are subjective; rather than objective. Furthermore, more emphasis should be on vigorous work-based assessment with sufficient formative feedback [9, 10]. 

Multiple tools such as DOPS (direct observation of procedural skills), mini-CEX, 360 degrees, and objective structured clinical examination (OSCE) are gaining popularity in medical education. Of the all mentioned options, DOPS and OSCE are excellent asset to evaluate medical competencies with immediate feedback. In the emergency room, the evidence shows that patient’s satisfaction and patient care will surge utilizing proper assessment methods [10-12].

The humanized approach to medicine is challenging for the education of medical candidates. The sociolegal approach to patients led to diminished patient contact and has urged us to change the education of the psychomotor (skill) domain of our medical residents. Each faculty and department should choose between the possible choices of the subsequent techniques. Utilizing all of them for the majority of the institutions is neither rational nor feasible [13]. 

The whole concept of the new methods is that the Orthodox teaching (transferring the skills incidentally from senior to junior) is not sufficient. Also, traditional learning of surgical techniques “see one, do one, and teach one” will threaten the patients' safety (Table 1) [14]. Simulation-based surgical tools have been widely propagated to decrease human error. Around one-tenth of preoperation patients will challenge with surgical complications. Using these methods will fascinatingly recreate many essential aspects of the real field [15-17]. 

Two overall categories are mechanical stimulator and virtual reality simulators (VRS). In the mechanical simulator, organs can be reached via surgical instruments. The learners start to learn laparoscopic skills and can practice on artificial or healthy tissues. A newer refined model to the traditional one is Lapsim virtual reality simulator that is more precise and has high fidelity [15-17]. VRS would be a great help to mastery the technical skills as it promotes upper limb dexterity. In the commercially available laparoscopy, virtual reality simulator will enable the trainees to grasp, cut, suture and improve real-time procedures [18].

In the past two decades, the role of gaming in education was a hub to a group of medical educationists, and both proponent and opponents of this view challenged considerably. Studies showed that gaming would enhance spatial memory and manual dexterity. Moreover, these are mainly cost-effective and exciting methods to students, while opponents believe that there are no well-established studies to address the long term adverse effect of videogames [19, 20].

**Table 1:** Advantages and disadvantages of the commercially available surgical training models [1].

No.	Surgical training model	Advantages	Disadvantages
1	Mechanical Simulator	• Reproducible • Standardized • Training of isolated skills • Cheap	• No high fidelity • No tissue rendering
2	Virtual reality Simulator	• Performance of real operations • Evaluation of isolated skills • Instant objective feedback	• Expensive • Questionable software and interface reliability
3	Animal lab	• Easy availability • Good tissue handling	• Ethical issues • Expensive
4	Cadaver lab	• High fidelity • Same anatomy (included individual variation) • No time pressure	• Ethical issues • Limited availability • Non-compliant bloodless tissues
5	Telementoring	• Exact anatomy • Realistic bleeding • Real OR setting • Independence of the first operator	• Requires another surgeon throughout surgery • The expert surgeon cannot operate directly
6	Telerobotic Assistance	• Exact anatomy • Realistic bleeding • Real OR setting	• Expensive • Requires another surgeon throughout surgery • Pressure of training

Apart from research, animal lab simulation is another considerable support for training and education. This method is highly accurate in comparison to other methods [21]. However, using the animals for such experimental learning should be based on considering legal and ethical issues and minimum possible harm to animals. Cadaveric lab simulation is a method of choice to teach surgical techniques. This method is highly useful in preparing residents before working on living bodies. In the case of specific surgeries, such as laparoscopic colorectal surgery, literature shows the superiority of this method on reality simulation. This method is also an excellent help for residents to have a realistic view of “anatomical variation of the human body” [22].

Telementoring is another valuable tool of surgical leaning, which provides remote guidance and help to perform the desired procedure via telecommunications technologies. It can cross any barrier and is highly supported to use in low developmental areas. This method has many benefits. Mentee and mentor can make a broad conversation on the case. Moreover, there is no need for the physical attendance of the mentor, which will decrease the cost. In a technical view, such innovative technology allows verbal communication between trainee and trainer and will promote self-stem of novice surgeon [23, 24].

Telerobotic manipulation and assistance are methods that the trainer can interfere in the operation. It is an authentic teaching method, although technically demanding, to educate in the real field. Both trainees and trainers have the same access to images and devices, while both of them are located outside of the surgical field [25]. In parallel to the education of surgical techniques, knowledge of clinical practice should be advocated. People who were born in the 20^th^ century were technology immigrants, while learners who belong to the current century are born and raised with technologies. Apart from the drawbacks of techniques, we believe that this trend is an excellent opportunity to advocate electronic learning in residency programs [26]. 

This shift of the curriculum cannot and should not be in an ad-hoc manner. So blended learning is required. Blended learning benefits from conventional teaching (interactive lecturing or a cased based discussion) and of electronic materials (podcasts-video and books) [26]. We wished to evaluate the potentials of our general residency program to implement the above-mentioned teaching methods. We designed and implemented a blended learning course on the primary survey for junior residents in our trauma center in Shiraz, Iran. Fortunately, the analysis of data showed that this global trend is also acceptable in our environment. The result of that study will be published soon. However, we call for more large-scale researches and implement these innovative approaches in the medical curricula.

## Conflict of Interest:

None declared.
